# Reinforcement Behavior of Chemically Unmodified Cellulose Nanofiber in Natural Rubber Nanocomposites

**DOI:** 10.3390/polym15051274

**Published:** 2023-03-02

**Authors:** Bunsita Wongvasana, Bencha Thongnuanchan, Abdulhakim Masa, Hiromu Saito, Tadamoto Sakai, Natinee Lopattananon

**Affiliations:** 1 Department of Rubber Technology and Polymer Science, Faculty of Science and Technology, Prince of Songkla University, Pattani 94000, Thailand; 2 Rubber Engineering & Technology Program, International College, Prince of Songkla University, Songkhla 90110, Thailand; 3 Department of Organic and Polymer Materials Chemistry, Tokyo University of Agriculture and Technology, Tokyo 184-8588, Japan; 4 Organization for Innovation & Social Collaboration, Shizuoka University, Shizuoka 432-8011, Japan

**Keywords:** natural rubber, nanocomposites, cellulose nanofibers, mechanical property, reinforcement

## Abstract

We investigated the reinforcement behavior of small amounts of chemically unmodified cellulose nanofiber (CNF) in eco-friendly natural rubber (NR) nanocomposites. For this purpose, NR nanocomposites filled with 1, 3, and 5 parts per hundred rubber (phr) of cellulose nanofiber (CNF) were prepared by a latex mixing method. By using TEM, a tensile test, DMA, WAXD, a bound rubber test, and gel content measurements, the effect of CNF concentration on the structure–property relationship and reinforcing mechanism of the CNF/NR nanocomposite was revealed. Increasing the content of CNF resulted in decreased dispersibility of the nanofiber in the NR matrix. It was found that the stress upturn in the stress–strain curves was remarkably enhanced when the NR was combined with 1–3 phr CNF, and a noticeable increase in tensile strength (an approximately 122% increase in tensile strength over that of NR) was observed without sacrificing the flexibility of the NR in the NR filled with 1 phr CNF, though no acceleration in their strain-induced crystallization was observed. Since the NR chains were not inserted in the uniformly dispersed CNF bundles, the reinforcement behavior by the small content of CNF might be attributed to the shear stress transfer at the CNF/NR interface through the interfacial interaction (i.e., physical entanglement) between the nano-dispersed CNFs and the NR chains. However, at a higher CNF filling content (5 phr), the CNFs formed micron-sized aggregates in the NR matrix, which significantly induced the local stress concentration and promoted strain-induced crystallization, causing a substantially increased modulus but reduced the strain at the rupture of the NR.

## 1. Introduction

Natural rubber (NR), a natural polymer of cis-1,4-polyisoprene obtained from natural sources, is an important raw material in the rubber industry. NR is known to have excellent mechanical properties due to its stretchable nature and its ability to crystallize after stretching [1,2]. It is, therefore, widely used in the rubber industry to manufacture rubber products, specifically, automobile tires, vibration insulators, and surgical gloves [3,4,5,6]. In the manufacturing process, NR is often added with fillers to achieve a desirable reinforcement, lower its price, and improve processability. 

Currently, the addition of cellulose nanofibers (CNFs) as a load-bearing filler has received significant attention for the formulation of high-performance polymer nanocomposites due to the outstanding mechanical properties presented by these CNFs. The CNFs were reported to exhibit high Young’s moduli (~100–160 GPa) [7,8,9,10,11] and high strength (~1.6–3 GPa) [12,13]. Due to the impressive mechanical properties of CNF, along with its inherent biodegradability, abundant availability, renewability, and low density, several research groups have investigated the use of CNF in a wide variety of polymers, such as thermosets/thermoplastics [14,15,16], biodegradable polymers [17,18,19], and synthetic rubbers [20,21,22,23]. Over the years, CNFs have become a potential nano-filler candidate to be combined with NR. Abraham et al. [24] dispersed CNF together with sulfur and zinc-based crosslinking agents in an NR matrix using an NR latex mixing method, and they found that the introduction of increasing CNF contents (1–10 wt% based on the weight of the dried NR) markedly increased the tensile modulus and strength of the NR. Similar observations have also been presented by other authors [25,26,27,28]. These authors have ascribed the properties increase in the NR to the establishment of a chemical network of Zn/cellulose nanofiber complex in the NR. Kato et al. [28] reported a great increase in the reinforcing efficiency of pristine CNFs in NR with increasing the filling level from 1 to 5 wt%. The use of chemically modified CNFs further increased the stiffness and reduced the thermal expansion of the NR nanocomposite due to the finely dispersed CNF and the formation of chemical crosslinks between the CNF and the NR. Owing to the above observations, the findings have clearly shown that both CNF dispersion and bonding strength at the interface between the CNF and the NR were the main reasons for the rise in mechanical and thermal properties at low levels of addition. 

Due to the stereoregularity of NR, the crystallization in NR under deformation, called strain-induced crystallization (SIC), presents a major interest in rubber technology. The formation of crystallites in a natural rubber network leads to a strengthening of this material, providing NR with a self-reinforcement character [29,30,31]. Generally, it is well-established that the strain-induced crystallization of NR is sensitive to the microstructure of the NR network and its changes during deformation [1,32]. Furthermore, the presence of popular nano-fillers such as nanoclay, silica, carbon black, carbon nanotubes, and graphene was found to activate an early crystallization, as well as promote the overall crystallization of NR during uniaxial deformation [4,5,33,34,35,36]. Recently, Wongvasana and co-workers [37] was the first group to compare the structure–property relationship of NR nanocomposites reinforced with nanoclay and CNF at a filling level of 5 phr. The results from this study showed clear distinctions between the nanoclay and the CNF in terms of their reinforcing effects and mechanisms. The nanoclays were found to finely disperse in the NR, and they effectively increased the crystalline phase in the NR due to the orientation of the NR chains introduced by the cooperation of the clay rotation and crosslinking in the NR network during stretching. As a consequence, the 5 phr nanoclay/NR nanocomposite exhibited high tensile strength and breaking strain. On the contrary, the CNF at a content of 5 phr formed an aggregated structure consisting of entangled nanofibers dispersed in the NR. The CNF aggregates were shown to impart high stiffness to the NR, with a low breaking strain. Interestingly, the ability of the aggregated CNFs to induce the NR crystallization upon stretching was also noted, even at low strain of approximately 150%. 

NR has shown different mechanical properties when combined with different loadings of fillers [4,38,39]. Previous works [4] have shown that the microstructure of NR was changed by the dispersed fillers and their contents, and the NR microstructure strongly affected the strain-induced crystallization and mechanical properties of the NR nanocomposites. Up to now, studies on the strain-induced crystallization of NR reinforced with CNF have been very limited, and therefore, information on the mechanistic reinforcement is not adequate for the development of eco-friendly and sustainable materials which require the effective use of CNFs. 

In this study, we aimed to explore CNF’s effects and the structures they form at different contents on the properties of NR. Pristine CNFs were used at concentrations of 1, 3, and 5 phr. The use of CNF without chemical modification is of benefit to manufacturing from an economical and environmental perspective. The CNFs were mixed with NR using a latex mixing method, as previously outlined in the literature [37], and crosslinked with dicumyl peroxide (DCP) to obtain CNF/NR nanocomposites. The neat NR was prepared and used as a control. To clarify the CNF’s effects on the mechanistic reinforcement of the NR at different contents, we investigated the microstructures, mechanical properties, bound rubber contents, crosslink densities, and strain-induced crystallization levels of the CNF/NR nanocomposites by transmission electron microscopy (TEM), tensile tests, dynamic mechanical analyses, measurements of bound rubber, solvent-induced swelling, and gel contents, and wide-angle X-ray diffraction (WAXD), respectively. 

## 2. Materials and Methods

### 2.1. Materials

High ammonia (HA) concentrated natural rubber (NR) latex containing a dry rubber content (DRC) of 60% was supplied by Yala Latex Co., Ltd. (Yala, Thailand). Cellulose nanofibers (CNF, Nanoforest-S) made from wood pulp using the aqueous counter collision (ACC) method were kindly supplied by Chuetsu Pulp and Paper Co., Ltd. (Tokyo, Japan). Dicumyl peroxide (DCP) was manufactured by Wuzhou International Co., Ltd. (Shenzhen, China), and 2, 2, 4-trimethyl-1,2-dihydroquinone (TMQ) was supplied by Lanxess AG (Cologne, Germany). Paraffinic oil (white oil grade A, no. 15) was provided by China Petrochemical International Co., Ltd. (Shanghai, China). 

### 2.2. Preparation of CNF/NR Nanocomposites

**Figure 1:**
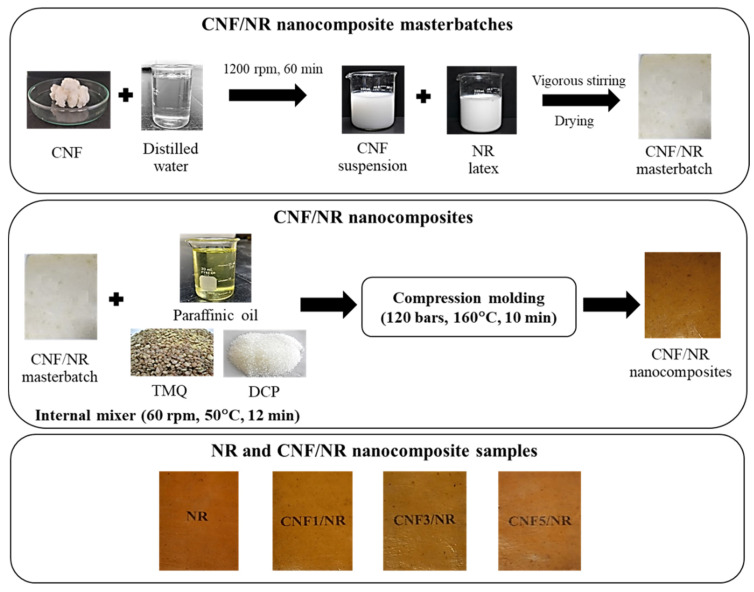
Schematic diagram of the preparation of the NR and the CNF/NR nanocomposites.

The CNF/NR nanocomposites were prepared through the latex mixing method schematically shown in Figure 1. In the latex mixing method, the aqueous CNF suspension (1 wt%), obtained by mixing the CNFs in water, as outlined in the literature [37], was firstly mixed with NR latex under vigorous stirring (600 rpm) at room temperature for 30 min using an IKA^®^ RW 20 digital mixer (IKA^®^-Werke, Staufen, Germany). The obtainable CNF/NR mixtures having amounts of CNF of 1, 3, and 5 phr were then dried at 50 °C for 2 days. The dried CNF/NR masterbatches were later compounded with the rubber additives in a Hakke internal mixer (Thermo Electron Corporation, Karlsruhe, Germany) at a temperature and rotor speed of 50 °C and 60 rpm, respectively, for 12 min. The compositions of the CNF/NR nanocomposite compounds are listed in Table 1. The compounded CNF/NR nanocomposites were crosslinked with DCP in a hot-pressing machine at 160 °C for 10 min. The neat NR used as a reference specimen was also prepared using the same procedure as described above. Photographs of the NR and NR nanocomposite samples are shown in Figure 1. The chemically unmodified CNF-reinforced NR was visibly transparent at CNF filling levels of 1–5 wt%. In this study, the DCP-crosslinked NR nanocomposites with 1, 3, and 5 phr CNF were designated CNF1/NR, CNF3/NR, and CNF5/NR, respectively. 

### 2.3. Characterization

#### 2.3.1. Transmission Electron Microscopy (TEM)

TEM was used to study the dispersion of the CNFs in the CNF/NR nanocomposites. TEM imaging was conducted using a JEOL JEM 2010 (JEOL Co., Tokyo, Japan). Ultra-thin sections (approximately 100 nm) were cut with a diamond knife at a temperature of −120 °C using an ultramicrotome (RMC MT-XL, RMC Products Group, Ventana Medical System, Inc., Oro Valley, AZ, USA).

#### 2.3.2. Wide-Angle X-ray Diffraction (WAXD) Measurements

The degree of crystallinity in the NR and the CNF/NR nanocomposites during tensile stretching was assessed by wide-angle X-ray diffraction (WAXD) using a NANO-Viewer system (Rigaku Co., Ltd., Tokyo, Japan). Cu-Kα radiation with a wavelength of 0.154 nm was generated at an accelerated voltage of 46 kV and a target current of 60 mA. The sample-to-detector distance was 15 mm. An imaging plate (IP) (Fujifilm BAS-SR 127) was used as a two-dimensional detector and an IP reading device (R-AXIS Ds3, Rigaku Co., Japan) was used to transform the obtained image to text data. The sample was stretched in steps after WAXD measurements at a fixed strain using a miniature tensile machine (Imoto Machinery Co., Ltd., Kyoto, Japan). The exposure time was 15 min at room temperature (20 °C). The scattering intensity was corrected with respect to the exposure time, the sample thickness, and the transmittance.

The area of the crystalline diffraction peaks assigned to the (200) and (120) planes and the area of the amorphous halo were fitted using Origin^®^9.1 software. The value of *X_c_* was calculated using Equation (1):

(1) $$X_{c}=\frac{A_{c}}{A_{c}+A_{a}}\times 100\;\%,$$

where *A_c_* represents the areas of the crystalline region and *A_a_* corresponds to the amorphous region.

#### 2.3.3. Mechanical Property Measurements

The mechanical properties were measured on a Hounsfield Tensometer (H10KS, Hounsfield Test Equipment Co., Ltd., Surrey, UK) at a temperature of 25 ± 2 °C with an extension rate of 500 ± 50 mm/min by ASTM D412. The dumb-bell-shaped specimens were cut from the crosslinked rubber films. An average of ten specimens was considered for the tensile test.

#### 2.3.4. Dynamic Mechanical Analysis (DMA)

The dynamic mechanical properties of the NR and the CNF/NR nanocomposites were measured using an advanced rheometric expansion system rheometer (model ARES-RDA W/FCO, TA Instruments Ltd., New Castle, DE, USA). The storage modulus (E′) and loss factor or damping factor (tan δ = E″/E′, where E″ is a loss modulus) were determined with the tension mode at temperatures ranging from −95 °C to 80 °C using a heating rate of 2 °C/min, a frequency of 1.0 Hz, and a dynamic strain amplitude of 0.5%.

#### 2.3.5. Bound Rubber

Bound rubber measurements were performed to determine the physical linkages between the rubber and the CNF. Approximately 0.2 g (g) of uncured rubber compounds contained in a metal cage were immersed in 20 mL of toluene at room temperature for 3 days, with the solvent replaced every day. Then, the samples were removed from the toluene solvent and dried at 105 °C until they reached a consistent weight. The bound rubber content was estimated using the following equation [40]:

(2) $$Bound\;rubber\;\left(\%\right)=\frac{W_{fg}-W_{f}}{W_{p}},$$

where *W_fg_* represents to the weighted sample after immersion, *W_f_* is the weight of the CNF in the specimen, and *W_p_* refers to the weight of the NR in the specimen.

#### 2.3.6. Gel Content

Gel content measurements were performed to measure the extent of the crosslinking of the NR phase in the NR and the CNF/NR nanocomposites. Specimens weighing between 0.17 and 0.20 g were cut into small pieces and directly immersed in a 250 mL round bottom boiling flask containing ~100 mL of toluene and attached to a condenser. The gel content determination was carried out for 8 h. The insoluble residues were taken out and dried at room temperature for 48 h prior to weighting. The gel content was calculated using the following equation [41]:

(3) $$Gel\;content\;=100-\left[\left(\frac{W_{final}}{\left(1-F\right)\;Wrubb}\right)\;\times \;100\right],$$

where *W_final_* is the weight of the sample after extraction, *W_rubb_* is the initial weight of the rubber in the sample, and *F* is the volume fraction of the filler.

## 3. Results and Discussion

### 3.1. Dispersion of CNF in the CNF/NR Nanocomposites

**Figure 2:**
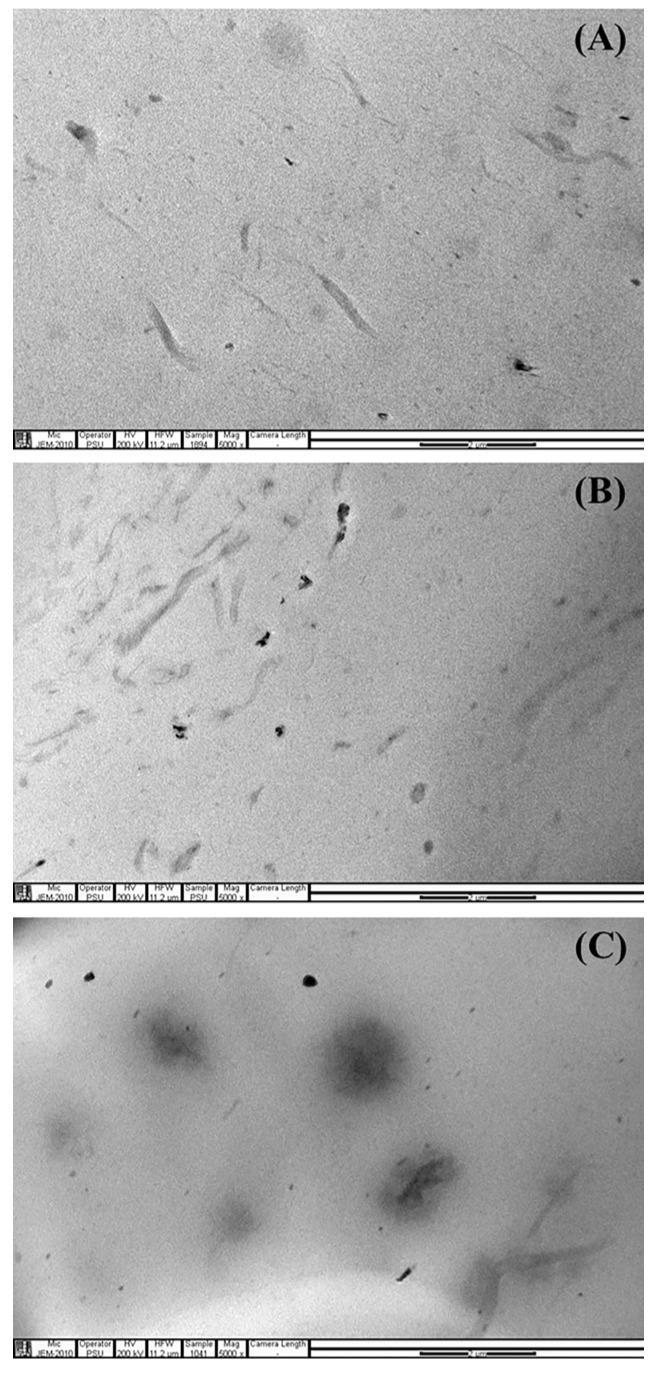
TEM images of (**A**) CNF1/NR, (**B**) CNF3/NR, and (**C**) CNF5/NR at low magnification (X5,000).

**Figure 3:**
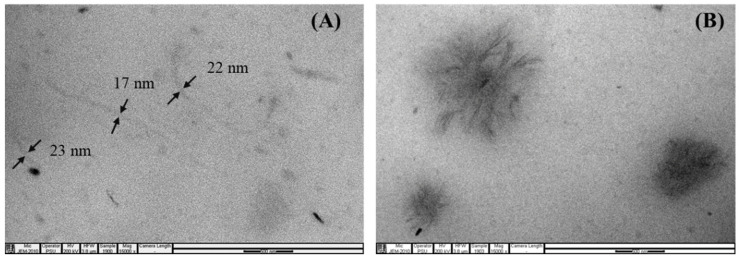
TEM images of (**A**) CNF1/NR and (**B**) CNF5/NR at high magnification (X15,000).

The effect of the CNF content on the filler dispersion state in the NR matrix was examined by the TEM technique, and the results are shown in Figure 2 and Figure 3. Figure 2 shows TEM photomicrographs of thin sections of the CNF/NRs containing 1, 3, and 5 phr CNF taken at low magnification levels. In the early work of Thomas et al. [25], in a TEM photograph of NR without filler, the absence of fillers was apparent. However, the obtained TEM images of the NR nanocomposites concerning the dispersion of the CNF showed the CNF structure in the NR matrix. The sizes of the CNFs in the various CNF/NR samples were measured from the TEM images using Image J software, and their sizes were represented by the thicknesses. The results are given in Table 2. From Figure 2A–C, it can be seen that different grades of CNF dispersion were formed in the NR matrixes, depending on the content of CNF. It has been reported that individual CNFs obtained from wood sources had thicknesses of approximately 3–5 nm [13,42,43]. Based on the measured sizes of the nanofibers shown in Figure 2 and Table 2, it was clear that the CNF1/NR consisted of CNFs which were separate from the nanofiber and bundles of nanofibers due to high extent of CNF-CNF interactions via the hydrogen bonding of the active hydroxyl group (-OH) on the CNF surfaces [14,22,44]. When the addition of the CNFs was increased to 3 phr, the nanofibers were held together to form fiber bundles, and their thicknesses were apparently increased (Figure 2B and Table 2). With further addition of CNFs of up to 5 phr, the CNFs were mostly aggregated, and the aggregated dimensions were approximately 1–3 µm (Figure 2C and Table 2). At higher magnification, as shown in Figure 3, the TEM images clearly displayed the nanofiber structure in the CNF1/NR sample and the aggregated structure composed of highly entangled nanofibers in the CNF5/NR sample. In an early work by Fiorote et al. [45], the effect of CNF content (0.5, 1, 2.5, and 5 phr) on the morphology of CNF/NR nanocomposites was investigated. The results showed that the degree of nanofiber dispersion decreased with increasing contents of CNF. Similarly, Zhang et al. [46] incorporated CNFs of different contents (1–10 phr) in NR nanocomposites, and they demonstrated that poor nanofiber dispersion was observed for the nanocomposites loaded with CNF in the amounts of 5 and 10 phr. In this study, the findings from the TEM analysis led to the conclusion that there was a homogeneously dispersed, nano-sized CNF in the CNF1/NR sample and a micro-sized domain of aggregated nanofiber in the CNF5/NR sample. 

### 3.2. Stress-Strain Behavior of NR and CNF/NR Nanocomposites 

**Figure 4:**
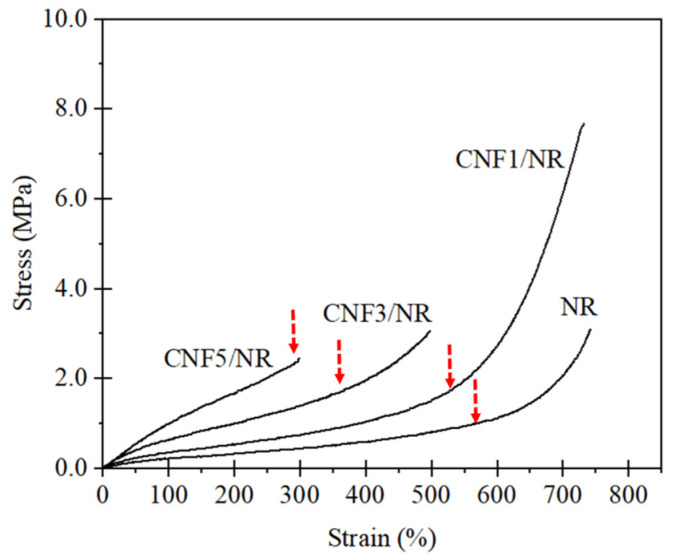
Stress–strain curves of the NR and the CNF/NR nanocomposites.

Figure 4 shows the representative stress–strain behavior of the CNF/NRs filled with different CNF contents. As can be seen in Figure 4, it was obvious that the characteristic stress–strain curves of the NR, CNF1/NR, and CNF3/NR samples, but not that of the CNF5/NR sample, were very similar; that is, their stresses gradually increased as a function of the applied strain and turned upward sharply beyond a certain strain, as indicated by the arrows. It was also interesting to see that the upward turn was pronounced upon the addition of the CNFs into the NR. In the unfilled NR, the abrupt upturn of stress at high strains was generally assigned to the strain-induced crystallization (SIC) process [4,47,48]. Conversely, the CNF5/NR sample showed a different stress–strain behavior. The tensile stress exerted on this sample was dramatically raised upon stretching until it reached the rupture stress at low applied strain (~300%), where the abrupt upturn in stress was about to occur. As we clearly demonstrated that the CNFs in the CNF5/NR sample were inhomogeneously dispersed in the NR (Figure 2 and Figure 3), the aggregated nanofibers in the CNF5/NR sample could have acted as crack precursors that reduced the breaking strain of the NR. 

The tensile moduli at 50%, 100%, and 300%, as well as the tensile strength, strain at break of the NR, and various CNF/NRs, were also compared, as shown in Table 3. These results clearly showed the influence of the different CNF addition levels on the mechanical properties of the NR nanocomposites. The tensile moduli at 50%, 100%, and 300% strains obviously increased with the increasing CNF content. Several authors have reported a dependence of the modulus of a polymer on the filler content [49,50,51]. In Table 3, it is seen that the increases in the moduli at the 50%, 100%, and 300% strains of the NR were significant in the CNF5/NR sample (the increases were 110%, 304%, and 420% for the 50% modulus, 100% modulus, and 300% modulus, respectively). The tensile strength of the CNF/NR samples increased when CNFs were incorporated at 1 phr, and then they leveled off as the CNF contents of 3–5 phr were added. For the CNF1/NR sample, it was seen that the tensile strength of the CNF1/NR sample was remarkably improved by approximately 122% over that of the NR, and its strain at break was approximately 757% comparable to that of the NR (which had a breaking strain of approximately 759%). The high tensile strength and good flexibility may be ascribed to the well-dispersed CNFs in the CNF1/NR sample. The crosslink density determined from the equilibrium swelling measurement is also included in Table 3. In general, the crosslink density of a composite material is a measure of the filler–rubber interaction [27,39]. Based on the data, it was clear that increments in overall crosslink density resulted from more interaction between the CNF and the NR. Therefore, the addition of more CNF caused higher restricted NR chain mobility, which accounted for the increase in the tensile modulus and the decrease in the rubber flexibility. However, the tensile strength was inconsistently increased with the increasing crosslink density.

Based on these observations, a noteworthy result obtained was that the characteristic stress–strain behaviors of the NR and the NR nanocomposites with lower CNF contents (1–3 phr CNF) were clearly distinguishable from those of the high CNF content samples (5 phr CNF). Furthermore, the tensile properties of the NR nanocomposites changed in variation with the incorporated CNF contents. To explain these observations, a study on the microstructural evolution of NR networks in various CNF/NR samples using WAXD analysis was carried out, and their features of strain-induced crystallization were compared and are discussed in the next section. 

### 3.3. Strain-Induced Crystallization of the NR and the CNF/NR Nanocomposites 

**Figure 5:**
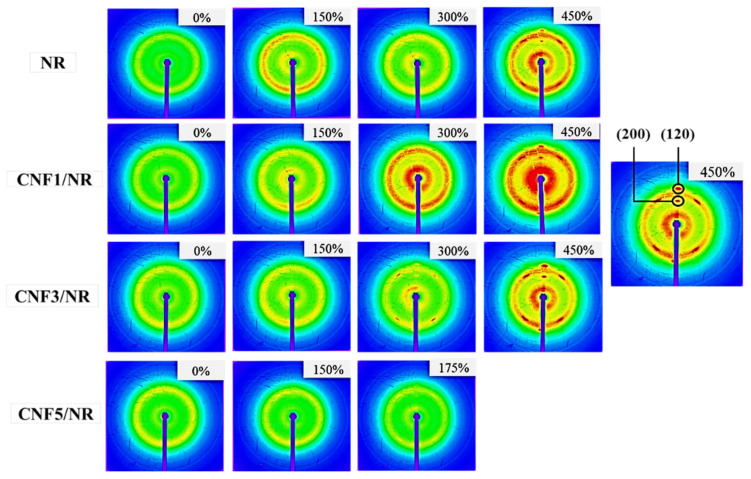
Typical two-dimensional WAXD images as a function of the applied strain for the NR and the CNF/NR nanocomposites.

Figure 5 displays two-dimensional (2D) WAXD images of the NR and the CNF/NR samples containing 1, 3, and 5 phr CNF at various applied strains. 

Figure 5 shows that the different positions of the reflection spots seen in these photographs were assigned to different crystallographic planes, and the crystallographic planes that corresponded to (200) and (120) were of interest. It was clear that the applied strain had a significant impact on the patterns in the WAXD images. At strains of 0 and 150%, no reflection spots were observed in these images due to the fact that no crystallization had occurred. On the other hand, several reflection spots belonging to different crystallographic planes appeared when the samples were stretched up to strains of approximately 175–300%. These reflection spots became more pronounced, with increasing deformations, suggesting that the strain promoted crystallization and molecular chain orientation [3]. 

**Figure 6:**
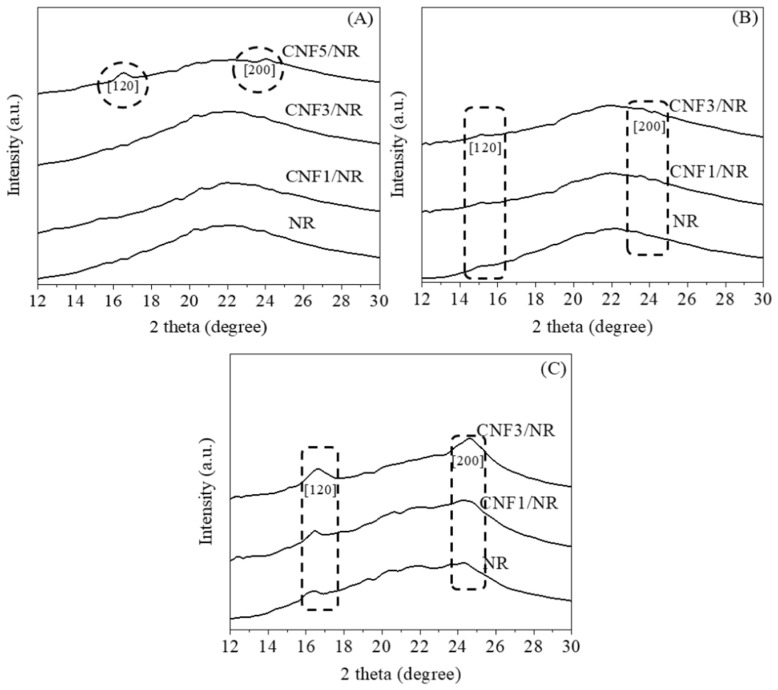
The 1D WAXD patterns of the NR and the CNF/NR nanocomposites measured at strains of (**A**) 200%, (**B**) 300%, and (**C**) 450%.

To obtain clear information about strain-induced crystallization in the CNF/NR samples, the 2D WAXD data were transformed into 1D data, and the results are shown in Figure 6. Figure 6 shows the 1D WAXD patterns of the NR and the various CNF/NR samples selected at strain levels of 200%, 300%, and 450%. The diffraction peaks observed at 2θ of approximately 16° and 24° corresponded to the (200) and (120) planes [52,53]. No crystal peaks were observed at 200% strains for the NR, CNF1/NR, and CNF3/NR samples, indicating crystallization had not occurred in these samples. The crystallization in the NR, CNF1/NR, and CNF3/NR samples was initially seen at a strain of 300%, in which the two diffraction peaks at 2θ of approximately 16° and 24° were observed. These two peaks became more pronounced with further deformation, implying the enhancement of the crystallinity with the strain. Unlike the NR and CNF/NR samples with 1–3 phr CNF, the diffraction peaks corresponding to the (200) and (120) planes in the CNF5/NR sample were observed at a low strain of 200%, suggesting an early crystallization process in this sample. Since the CNF5/NR sample was broken at strain of approximately 300%, no further enhancement of crystallinity was observed in this sample. 

**Figure 7:**
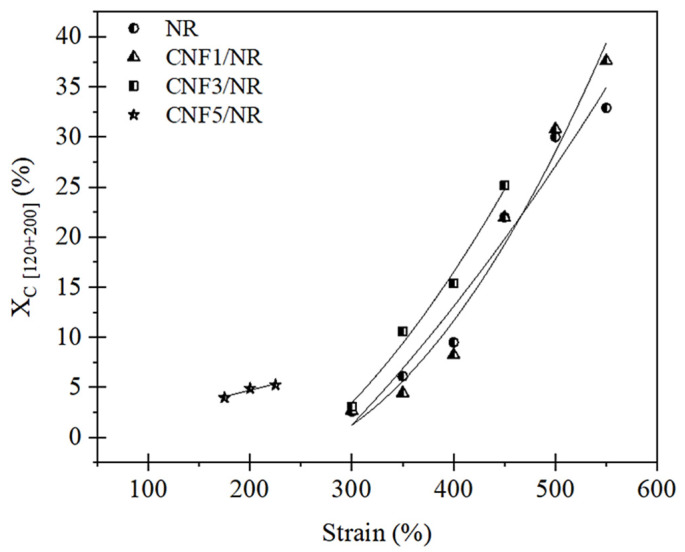
Degree of crystallinity (*X_c_*) as a function of the applied strain for the NR and the CNF/NR nanocomposites.

Based on the 1D WAXD images, the crystallinity (*X_c_*) of the stretched NR and different CNF/NR samples could be estimated using Equation (1). The *X_c_* results are shown in Figure 7.

Figure 7 shows the change in crystallinity degree (*X_c_*) as a function of the applied strain for the NR and the various CNF/NR samples filled with different amounts of CNFs. It was obvious that the *X_c_* of all samples increased with the increasing strain, indicating that the crystallization of the NR and the nanocomposites was caused by tensile deformations. The *X_c_* values of the NR and the CNF1/NR and CNF3/NR samples were initially seen at a strain of approximately 300%. This implied that the onset strains of the strain-induced crystallization in these three samples were similar. The variation in *X_c_* upon stretching and at the same strain levels was also comparable among these samples, suggesting that the crystallization process that took place in the NR was similar to those of the CNF1/NR and CNF3/NR samples, even though the latter contained CNF as reinforcement. Therefore, the characteristic patterns of the stress–strain curves of the NR and the CNF/NR samples containing 1 and 3 phr CNF were very similar, as discussed earlier (Figure 4). On the other hand, the CNF5/NR sample showed a dramatic decrease in strain value (175%) at the onset of crystallization and a progression of crystallization with increasing the applied strain from 175% to 225%. No further deformation and crystallization developed because the sample had failed (~300% strain). It was proposed that the immobilized NR chains at the surface of the aggregated CNF contributed to the local stress concentration and the strain-induced crystallization behavior in the CNF5/NR sample [37], and thereby, they significantly increased the moduli at different strains (Figure 4 and Table 3). As the CNF5/NR sample was strained up to approximately 300%, the amount of local stress concentration was significantly high, which resulted in the quick failure of the CNF5/NR sample. 

The most surprising aspect of the above observations was that the accelerated straininduced crystallization was not detected in the CNF/NR samples with comparatively lower CNF contents (1–3 phr), and their degrees of crystallization upon stretching did not depend on their CNF content, though the tensile properties showed different variations. Thus, further investigations to reveal the influence of CNF concentration on the nanocomposite structure and their reinforcement effects through DMA analysis, bound rubber formation, and gel content measurement were performed. 

### 3.4. Dynamic Mechanical Properties of the CNF/NR Nanocomposites

**Figure 8:**
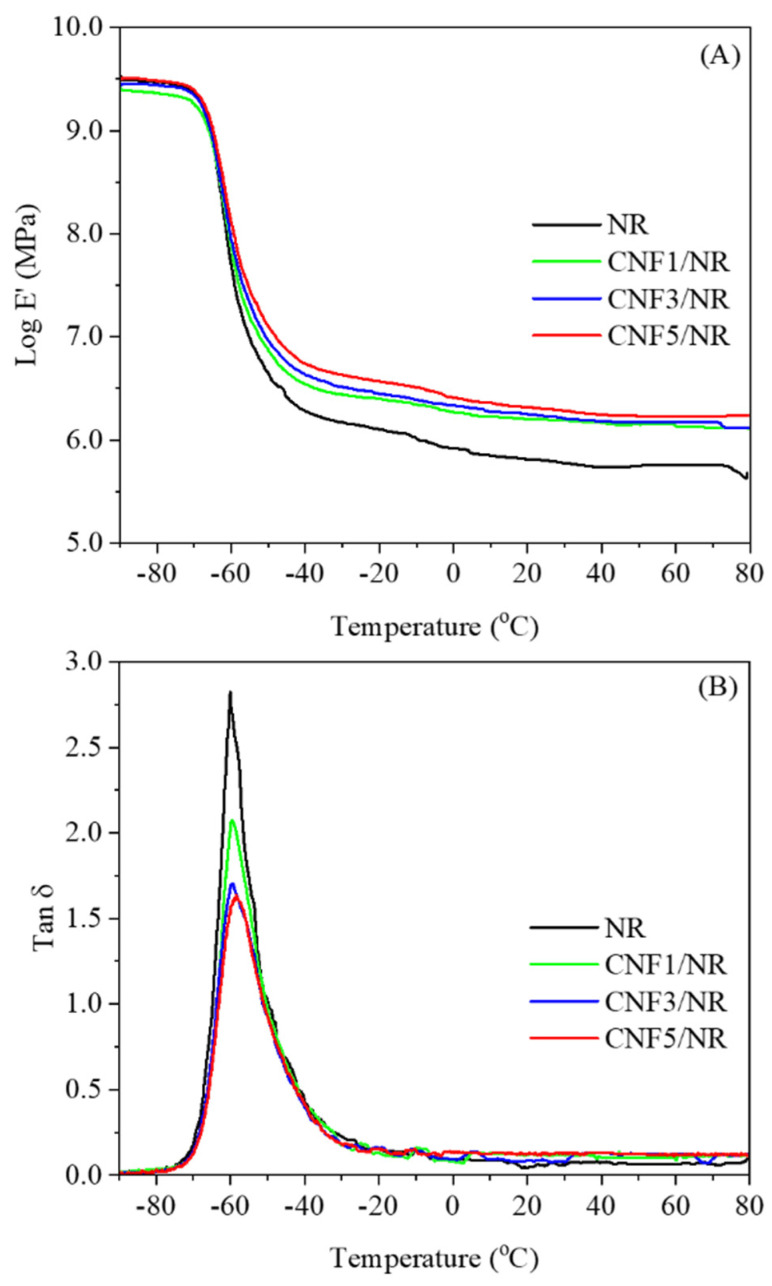
Variations in (**A**) storage modulus (log E′) and (**B**) tan δ as a function of temperature for the NR and the CNF/NR nanocomposites.

Figure 8 shows the correlation between the storage modulus (E′) and the damping factor (Tan δ) as a function of the temperature for the NR and the CNF/NR samples containing 1, 3, and 5 phr CNF. Generally, the addition of CNF significantly enhanced the E′ in a rubbery state and decreased the tan δ, reflecting the influence of CNF on the reinforcement of the NR. The values of E′ at 25 °C, the tan δ_max_ of the NR (the height of the tan δ peak), and the glass transition temperature (T_g_) of the NR and the CNF/NR samples are also listed for comparison in Table 4. 

As can be seen from Figure 8A and Table 4, the inclusion of CNF improved the E′ of the NR at 25 °C, and the magnitude of the increment increased with increasing CNF contents. This resulted from the rubber being more rigid as a result of the higher filling levels of CNF [37]. The rigidity of the pristine CNF could impede the movement of the chain segment of the NR through the filler–rubber interfacial actions [14,54]. Thus, in our study, it was likely that that the improvement in the E′ at 25 °C could mainly attributed to the physical interaction or entanglement between the pristine CNFs and the NR chains in the CNF/NR samples. Moreover, it was seen that the pristine CNFs reduced the tan δ_max_ of the NR depending on the amount of CNF. The reduction in the tan δ_max_ with the increasing CNF contents indicated the higher restricted movement of the NR chain segments at the interface of the CNF and the NR [37,55,56,57]. The glass transition temperature illustrated by the tan δ peak temperature of the NR (−60.1 °C) was systematically shifted to higher temperature as the CNF content was increased. When the NR chains adhered to the surfaces of CNFs via interfacial interactions, as discussed previously, a higher energy was required to achieve the same level of chain segment movement in the CNF/NR samples than in the neat NR. Similar results have been found in CNF-reinforced polyethylene oxide (PEO) [14] and styrene-butadiene (SBR) nanocomposites [22]. Therefore, the lowering of the tan δ_max_ and the increment of the T_g_ with the incorporated CNF further substantiated the interfacial interaction between the nanofibers and the NR at the interface of the CNF/NR samples. Owing to the results demonstrated by the DMA technique, the CNF-reinforced NR nanocomposites showed better dynamic properties than the NR due to the interfacial reinforcement in the CNF/NR nanocomposites.

### 3.5. Bound Rubber and Gel Content of the CNF/NR Nanocomposites

Table 5 shows the effect of CNF concentration on bound rubber and gel content formation. The bound rubber is a measure of the elastomer adsorption onto the filler surface [40,58], while the gel content reveals information about the chemical crosslink density in the NR network [59]. 

It was seen that bound rubber was not detected in the CNF1/NR and CNF3/NR samples. This implied that the NR molecules did not interact chemically with the reinforcing nanofibers and they could be readily removed from the unreacted CNF1/NR and CNF3/NR compounds after being immersed in toluene for a given period of time. On the other hand, the CNF5/NR sample in which the nanofibers were mostly aggregated (Figure 2C and Figure 3B) showed a significant bound rubber content of approximately 9.06%. It was shown that the non-extractable NR observed in the CNF5/NR sample was formed by the insertion of NR chains into the aggregated CNFs. These inserted NR chains led to a number of immobilized NR chains and a significant local stress concentration, which had a large influence on the tensile properties and crystalline formation in the CNF5/NR sample, as discussed earlier in our previous work [37]. These results suggested that the NR chains were not inserted into the CNF bundles of the CNF1/NR and CNF3/NR samples.

Considering the data of gel content measurements in Table 5, it was clearly seen that each gel content of the NR and the CNF/NR samples filled with 1, 3, and 5 CNF phr was not different, meaning that the incorporation of CNF did not change the degree of chemical crosslinking in the NR by the peroxide vulcanization. Therefore, the changes in the mechanical properties of the CNF/NR nanocomposites were largely governed by the CNFs’ dispersibility and their microstructure formations. Unlike the CNF5/NR sample, the NR nanocomposites reinforced with relatively lower CNF contents, particularly the CNF1/NR sample, showed high levels of improvement in the tensile strength of the NR, with good flexibility, even though the acceleration of the strain-induced crystallization by the CNF incorporation and the bound rubber in this sample were not observed. These results may interestingly suggest a different reinforcement mechanism of the CNFs in the NR nanocomposites with relatively low (1 phr) and high (5 phr) CNF contents.

### 3.6. Model of Reinforcement Mechanism

**Figure 9:**
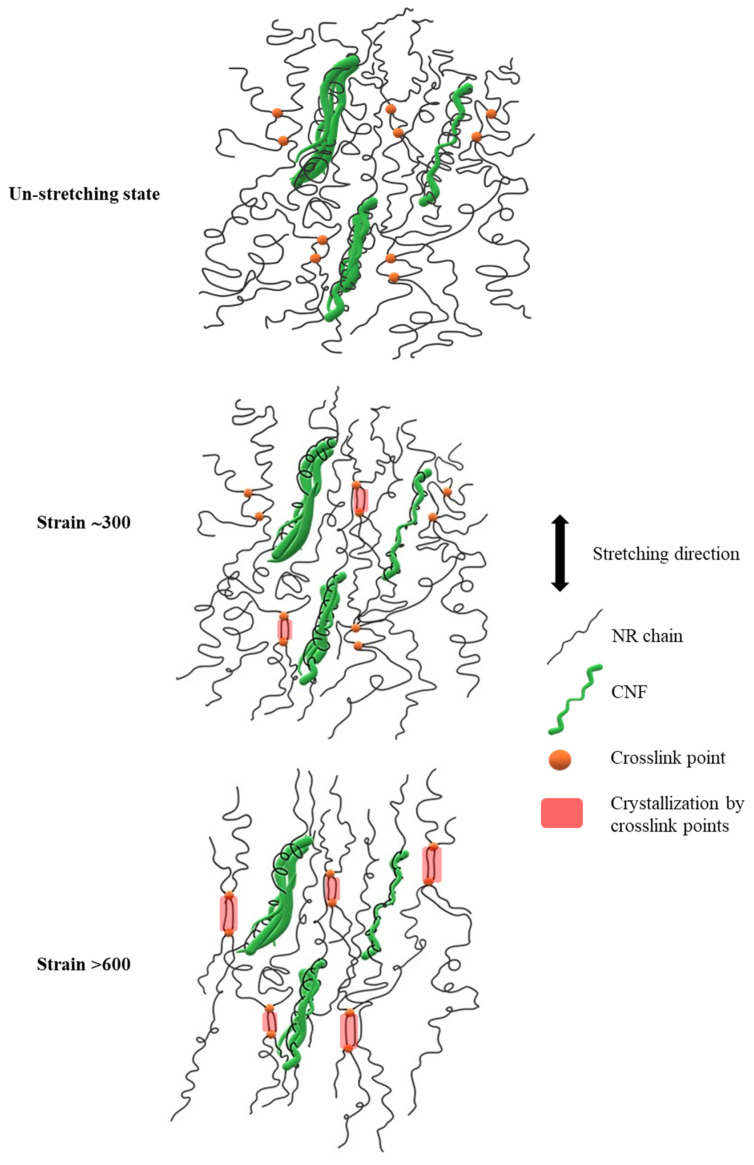
Proposed model for the strain-induced crystallization mechanism of the CNF/NR nanocomposite with 1 phr CNF.

Based on the observations mentioned above, we proposed a mechanistic model explaining the reinforcement of the CNF/NR nanocomposites with low CNF contents, as depicted in Figure 9. The focus was on the NR nanocomposites filled with 1 phr CNF, as the reinforcement mechanism of the NR nanocomposites containing high CNF loading (5 phr) was well-described in our earlier publication [37]. It should be noted here that the CNF1/NR sample exhibited separate nanofibers and small bundles of a nano-sized scale (Figure 2(A)), implying that the surface area of the CNF for the interaction with the NR in this sample was relatively high. 

In an unstretched state, the long chains of the NR molecules would most likely interact with the single CNF and bundled CNFs through physical entanglement, as shown in Figure 9. Upon tensile stretching, the NR network was deformed, whereas the stiff CNF was not deformed. Theoretically, in a classical model of short-fiber composites, the reinforcement of rigid fiber occurs through the transfer of tensile stress from the matrix to the fiber by means of interfacial shear stress [60,61]. By this mechanism, the tensile stress in the NR was built up by the transfer of the shear stress from the NR to the CNF across the CNF/NR interface. Therefore, the CNF in the CNF1/NR would contribute to carry more tensile stress upon deformation, owing to relatively large interfacial area for the stress transfer from the CNF to the NR. However, the nano-sized CNFs prevented the NR chains from aligning and crystallizing because of the lack of stress concentration at the interface between the CNF and the NR chains in the CNF1/NR sample. As a result, enhancement of the strain-induced crystallization caused by the nanofiber was not observed in the NR nanocomposites containing small amounts of CNF. On the other hand, the presence of the local stress concentration at the interface between the aggregated CNF and the NR caused by the mutually entangled structure of the CNF aggregates and the NR chains, as demonstrated by the bound rubber measurements (Table 5), was the main factor for the acceleration of the strain-induced crystallization at the low strain in the CNF5/NR sample (Figure 5, Figure 6 and Figure 7). When the tensile deformation reached a strain of 300%, crystallization was observed in the CNF1/NR sample, which was due to the strain-induced crystallization by the short NR chains around the dense crosslinking points. The crystallization of the NR matrix progressively increased with the applied strains because the strain caused the orientation and alignment of the NR chains. At a large tensile deformation (>600% strain), the interfacial shear stress at the interface region between the CNF and the NR was significantly high, leading to a large increase in load bearing in the CNF and, thus, a significant enhancement of the NR reinforcement. The breaking strain of CNF1/NR was also comparable to the neat NR owing to the stretching without debonding at the CNF1/NR interface by the interaction through the physical entanglement.

## 4. Conclusions

We found the reinforcement behaviors of small amounts of chemically unmodified cellulose nanofiber (CNF) in eco-friendly natural rubber (NR). The tensile modulus and the storage modulus of the CNF-reinforced NR increased with increasing CNF concentrations. The NR nanocomposite with 1 phr CNF showed the maximum tensile strength, which was an approximate 122% increase over that of the NR, together with a large strain at break (757%). The CNF in amounts of 1–3 phr were well-dispersed in the NR matrixes, without microscaled aggregation, leading to significant enhancements in stress upturn during stretching. However, it was observed that the addition of CNF at low concentrations (1–3 phr) did not participate in the strain-induced crystallization process of the NR, and their degree of crystallinity was not dependent on the CNF filling contents. Therefore, the high tensile strength for the 1 phr CNF-filled NR nanocomposite was based on the increase in the dispersion state of the CNF, which, in turn, increased the CNF/NR interaction for the effective stress transfer capability from the NR to the embedded CNF. On the other hand, at a filling content of 5 phr CNF, the nanofibers were aggregated, resulting in a local stress concentration and accelerated strain-induced crystallization. This contributed to a high tensile modulus but low tensile strength and strain at break. Thus, this study revealed that the effects of CNF on the mechanistic reinforcement of NR varied depending on the different CNF filling concentrations.

## Figures and Tables

**Table 1:** Formulation of the NR and the CNF/NR nanocomposites.

Ingredients	Parts per Hundred Rubber (phr)			
	NR	CNF1/NR	CNF3/NR	CNF5/NR
NR	100	100	100	100
CNF	-	1	3	5
Paraffinic oil	20	20	20	20
TMQ	2	2	2	2
DCP	1	1	1	1

**Table 2:** Dimensions of the dispersed CNFs in the CNF/NR nanocomposites.

Samples	Dimension Range of CNFs (nm)	Average Thickness of the CNFs (nm)
CNF1/NR	3–184	65 ± 63
CNF3/NR	30–345	140 ± 99
CNF5/NR	1000–3000	1700 ± 700

**Table 3:** Summary of the mechanical properties of the NR and the CNF/NR nanocomposites.

Samples	50% Modulus (MPa)	100% Modulus (MPa)	300% Modulus (MPa)	Tensile Strength (MPa)	Elongation at Break (%)	Crosslink Density (×10^−5^ mol/g)
NR	0.22 ± 0.02	0.24 ± 0.03	0.49 ± 0.06	3.26 ± 0.66	759 ± 20	3.22 ± 0.20
CNF1/NR	0.23 ± 0.03	0.35 ± 0.02	0.70 ± 0.06	7.26 ± 1.03	757 ± 38	3.67 ± 0.14
CNF3/NR	0.42 ± 0.05	0.76 ± 0.08	1.56 ± 0.16	3.08 ± 0.47	470 ± 43	4.79 ± 0.20
CNF5/NR	0.50 ± 0.06	0.90 ± 0.03	2.55 ± 0.43	2.56 ± 0.31	302 ± 21	4.92 ± 0.11

**Table 4:** Storage moduli (log E′) at 25 °C, maximum tan δ peaks (tan δ_max_), and glass transition temperatures (T_g_) of the NR and the CNF/NR nanocomposites.

Samples	Log E′ at 25 °C (MPa)	Tan δ _max_	T_g_ (°C)
NR	5.79	2.85	−60.1
CNF1/NR	6.18	2.55	−59.2
CNF3/NR	6.21	1.71	−58.9
CNF5/NR	6.30	1.64	−58.1

**Table 5:** Bound rubber contents and gel contents of the NR and the CNF/NR nanocomposites.

Samples	Bound Rubber Content (%)	Gel Content (%)
NR	N/A	80.12 ± 0.11
CNF1/NR	N/A	80.24 ± 0.32
CNF3/NR	N/A	80.38 ± 0.08
CNF5/NR	9.06 ± 1.18	80.43 ± 0.73

## Data Availability

The data presented in this study are available on request from the corresponding author.

## References

[1] Toki S., Sics I., Ran S., Liu L., Hsiao B.S. (2003). Molecular orientation and structural development in vulcanized polyisoprene rubbers during uniaxial deformation by in situ synchrotron X-ray diffraction. Polymer.

[2] Trabelsi S., Albouy P.-A., Rault J. (2003). Crystallization and Melting Processes in Vulcanized Stretched Natural Rubber. Macromolecules.

[3] Masa A., Iimori S., Saito R., Saito H., Sakai T., Kaesaman A., Lopattananon N. (2015). Strain-induced crystallization behavior of phenolic resin crosslinked natural rubber/clay nanocomposites. J. Appl. Polym. Sci..

[4] Masa A., Saito R., Saito H., Sakai T., Kaesaman A., Lopattananon N. (2016). Phenolic resin-crosslinked natural rubber/clay nanocomposites: Influence of clay loading and interfacial adhesion on strain-induced crystallization behavior. J. Appl. Polym. Sci..

[5] Masa A., Saito H., Sakai T., Kaesaman A., Lopattananon N. (2017). Morphological evolution and mechanical property enhancement of natural rubber/polypropylene blend through compatibilization by nanoclay. J. Appl. Polym. Sci..

[6] Nie Y., Qu L., Huang G., Wang X., Weng G., Wu J. (2014). Homogenization of Natural Rubber Network Induced by Nanoclay. J. Appl. Polym. Sci..

[7] Hsieh Y.-C., Yano H., Nogi M., Eichhorn S.J. (2008). An estimation of the Young’s modulus of bacterial cellulose filaments. Cellulose.

[8] Rusli R., Eichhorn S.J. (2008). Determination of the stiffness of cellulose nanowhiskers and the fiber-matrix interface in a nanocomposite using Raman spectroscopy. Appl. Phys. Lett..

[9] Matsuo M., Sawatari C., Iwai Y., Ozaki F. (1990). Effect of Orientation Distribution and Crystallinity on the Measurement by X-ray Diffraction of the Crystal Lattice Moduli of Cellulose I and II. Macromolecules.

[10] Sakurada I., Nukushina Y., Ito T. (1962). Experimental determination of the elastic modulus of crystalline regions in oriented polymers. J. Polym. Sci..

[11] Šturcová A., Davies G.R., Eichhorn S.J. (2005). Elastic Modulus and Stress-Transfer Properties of Tunicate Cellulose Whiskers. Biomacromolecules.

[12] Saito T., Kuramae R., Wohlert J., Berglund L.-A., Isogai A. (2013). An Ultrastrong Nanofibrillar Biomaterial: The Strength of SingleCellulose Nanofibrils Revealed via Sonication-Induced Fragmentation. Biomacromolecules.

[13] Nechyporchuk O., Belgacem M.N., Bras J. (2016). Production of cellulose nanofibrils: A review of recent advances. Ind. Crop. Prod..

[14] Xu X., Liu F., Jiang L., Zhu J.Y., Haagenson D., Wiesenborn D.P. (2013). Cellulose Nanocrystals vs. Cellulose Nanofibrils: A Comparative Study on Their Microstructures and Effects as Polymer Reinforcing Agents. ACS Appl. Mater. Interfaces.

[15] Wang G., Yang X., Wang W. (2019). Reinforcing Linear Low-Density Polyethylene with Surfactant-Treated Microfibrillated Cellulose. Polymers.

[16] Yasim-Anuar T.A.T., Arin H., Norrrahim M.N.F., Hassan M.A., Andou Y., Tsukegi T., Nishida H. (2020). Well-Dispersed Cellulose Nanofiber in Low Density Polyethylene Nanocomposite by Liquid-Assisted Extrusion. Polymers.

[17] Siqueira G., Bras J., Dufresne A. (2009). Cellulose whiskers versus microfibrils: Influence of the nature of the nanoparticle and its surface functionalization on the thermal and mechanical properties of nanocomposites. Biomacromolecules.

[18] Herrera N., Mathew A.P., Oksman K. (2015). Plasticized polylactic acid/cellulose nanocomposites prepared using melt-extrusion and liquid feeding: Mechanical, thermal and optical properties. Compos. Sci. Technol..

[19] Lo Re G., Engström J., Wu Q., Malmström E., Gedde U.W., Olsson R.T., Berglund L. (2018). Improved Cellulose Nanofibril Dispersion in Melt-Processed Polycaprolactone Nanocomposites by a Latex-Mediated Interphase and Wet Feeding as LDPE Alternative. ACS Appl. Nano Mater..

[20] Fumagalli M., Berriot J., Gaudemaris B., Veyland A., Putaux J.-L., Molina-Boisseau S., Heux L. (2018). Rubber Materials from Elastomers and Nanocellulose Powders: Filler Dispersion and Mechanical Reinforcement. Soft Matter.

[21] Fukui S., Ito T., Saito T., Noguchi T., Isogai A. (2019). Surface-hydrophobized TEMPO-nanocellulose/rubber composite films prepared in heterogeneous and homogeneous systems. Cellulose.

[22] Sinclair A., Zhou X., Tangpong S., Bajwa D.S., Quadir M., Jiang L. (2019). High-Performance Styrene-Butadiene Rubber Nanocomposites Reinforced by Surface-Modified Cellulose Nanofibers. ACS Omega.

[23] Balachandrakurup V., Gopalakrishnan J. (2022). Enhanced performance of cellulose nanofibre reinforced styrene butadiene rubber nanocomposites modified with epoxidised natural rubber. Ind. Crop. Prod..

[24] Abraham E., Deepa B., Pothan L.A., John M., Narine S.S., Thomas S., Anandjiwala R. (2013). Physicomechanical properties of nanocomposites based on cellulose nanofibre and natural rubber latex. Cellulose.

[25] Thomas M.G., Abraham E., Jyotishkumar P., Maria H.J., Pothan L.A., Thomas S. (2015). Nanocelluloses from jute fibres and their nanocomposites with natural rubber: Preparation and characterization. Int. J. Biol. Macromol..

[26] Kumagai A., Tajima N., Iwamoto S., Morimoto T., Nagatani A., Okazaki T., Endo T. (2019). Properties of natural rubber reinforced with cellulose nanofibers based on fiber diameter distribution as estimated by differential centrifugal sedimentation. Int. J. Biol. Macromol..

[27] Dominic M., Joseph R., Begum P.M.S., Joseph M., Padmanabhan D., Morris L.A., Kumar A.S., Formela K. (2020). Cellulose Nanofibers Isolated from the Cuscuta Reflexa Plant as a Green Reinforcement of Natural Rubber. Polymers.

[28] Kato H., Nakatsubo F., Abe K., Yano H. (2015). Crosslinking via sulfur vulcanization of natural rubber and cellulose nanofibers incorporating unsaturated fatty acids. RSC Adv..

[29] Chenal J.-M., Gauthier C., Chazeau L., Guy L., Bomal Y. (2007). Parameters governing strain induced crystallization in filled natural rubber. Polymer.

[30] Laghmach R., Biben T., Chazeau L., Chenal J.M., Munch E., Gauthier C., Gil-Negrete N., Alonso A. (2013). Strain-induced crystallization in natural rubber: A model for the microstructural evolution. Constitutive Models for Rubber VIII.

[31] Candau N., Laghmach R., Chazeau L., Chenal J.-M., Gauthier C., Biben T., Munch E. (2014). Strain-Induced Crystallization of Natural Rubber and Cross-Link Densities Heterogeneities. Macromolecules.

[32] Masa A., Hayeemasae N., Soontaranon S., Mohd Pisal M.H., Mohamad Rasidi M.S. (2021). Effect of Stretching Rate on Tensile Response and Crystallization Behavior of Crosslinked Natural Rubber. Malays. J. Fundam. Appl. Sci..

[33] Fu X., Huang G., Xie Z., Xing W. (2015). New insights into reinforcement mechanism of nanoclay-filled isoprene rubber during uniaxial deformation by in situ synchrotron X-ray diffraction. RSC Adv..

[34] Ozbas B., Toki S., Hsiao B.S., Chu B., Register R.A., Aksay I.A., Prud’homme R.K., Adamson D.H. (2012). Strain-Induced Crystallization and Mechanical Properties of Functionalized Graphene Sheet-Filled Natural Rubber. J. Polym. Sci. B Polym. Phys..

[35] Beurrot-Borgarino S., Huneau B., Verron E., Rublon P. (2013). Strain-induced crystallization of carbon black-filled natural rubber during fatigue measured by in situ synchrotron X-ray diffraction. Int. J. Fatigue.

[36] Weng G., Huang G., Qu L., Nie Y., Wu J. (2010). Large-Scale Orientation in a Vulcanized Stretched Natural Rubber Network: Proved by In Situ Synchrotron X-ray Diffraction Characterization. J. Phys. Chem. B.

[37] Wongvasana B., Thongnuanchan B., Masa A., Saito H., Sakai T., Lopattananon N. (2022). Comparative Structure–Property Relationship between Nanoclay and Cellulose Nanofiber Reinforced Natural Rubber Nanocomposites. Polymers.

[38] Arroyo M., Lo’pez-Manchado M.A., Herrero B. (2003). Organo-montmorillonite as substitute of carbon black in natural rubber compounds. Polymer.

[39] Qu L., Huang G., Liu Z., Zhang P., Weng G., Nie Y. (2009). Remarkable reinforcement of natural rubber by deformation-induced crystallization in the presence of organophilic montmorillonite. Acta Mater..

[40] Dannenberg E.M. (1986). Bound Rubber and Carbon Black Reinforcement. Rubber Chem. Technol..

[41] Lopattananon N., Tanglakwaraskul S., Kaesaman A., Seadan M., Sakai T. (2014). Effect of Nanoclay Addition on Morphology and Elastomeric Properties of Dynamically Vulcanized Natural Rubber/Polypropylene Nanocomposites. Int. Polym. Process..

[42] Siró I., Plackett D. (2010). Microfibrillated cellulose and new nanocomposite materials: A review. Cellulose.

[43] Mishra R.K., Sabu A., Tiwari S.K. (2018). Materials chemistry and the futurist eco-friendly applications of nanocellulose: Status and prospect. J. Saudi Chem. Soc..

[44] Kargarzadeh H., Mariano M., Gopakumar D., Ahmad I., Thomas S., Dufresne A., Huang J., Lin N. (2018). Advances in cellulose nanomaterials. Cellulose.

[45] Fiorote J.A., Freire A.P., Rodrigues D.D.S., Martins M.A., Andreani L., Valadares L.F. (2019). Preparation of composites from natural rubber and oil palm empty fruit bunch cellulose: Effect of cellulose morphology on properties. Bioresources.

[46] Zhang C., Zhai T., Sabo R., Clemons C., Dan Y., Turng L.-S. (2014). Reinforcing Natural Rubber with Cellulose Nanofibrils Extracted from Bleached Eucalyptus Kraft Pulp. J. Biobased Mater. Bioenergy.

[47] Thomas S., Stephen R. (2010). Rubber Nanocomposites: Preparation, Properties and Applications.

[48] Karino T., Ikeda Y., Yasuda Y., Kohjiya S., Shibayama M. (2007). Nonuniformity in Natural Rubber As Revealed by Small-Angle Neutron Scattering, Small-Angle X-ray Scattering, and Atomic Force Microscopy. Biomacromolecules.

[49] Dalmas F., Chazeau L., Gauthier C., Cavaillé J.-Y., Dendievel R. (2006). Large deformation mechanical behavior of flexible nanofiber filled polymer nanocomposites. Polymer.

[50] Kristo E., Biliaderis C.G. (2007). Physical properties of starch nanocrystal-reinforced pullulan films. Carbohydr. Polym..

[51] Georgopoulos S., Tarantili P.A., Avgerinos E., Andreopoulos A.G., Koukios E.G. (2005). Thermoplastic polymers reinforced with fibrous agricultural residues. Polym. Degrad. Stab..

[52] Beurrot-Borgarino S., Huneau B., Verron E., Thiaudière D., Mocuta C., Zozulya A. (2014). Characteristics of Strain-Induced Crystallization in Natural Rubber During Fatigue Testing: In situ Wide-Angle X-ray Diffraction Measurements Using Synchrotron Radiation. Rubb. Chem. Technol..

[53] French A.D. (2014). Idealized powder diffraction patterns for cellulose polymorphs. Cellulose.

[54] Peng S., Iroh J.O. (2022). Dependence of the Dynamic Mechanical Properties and Structure of Polyurethane-Clay Nanocomposites on the Weight Fraction of Clay. Compos. Sci..

[55] Visakh P.M., Thomas S., Oksman K., Mathew A.P. (2012). Effect of cellulose nanofibers isolated from bamboo pulp residue on vulcanized natural rubber. BioRes.

[56] Ikeda Y., Phakkeeree T., Junkong P., Yokohama H., Phinyocheep P., Kitano R., Kato A. (2017). Reinforcing biofiller “Lignin” for high performance green natural rubber nanocomposites. RSC Adv..

[57] Kumar V., Alam M.N., Manikkavel A., Song M., Lee D.-J., Park S.-S. (2021). Silicone Rubber Composites Reinforced by Carbon Nanofillers and Their Hybrids for Various Applications: A Review. Polymers.

[58] Robertson C.G., Hardman N.J. (2021). Nature of Carbon Black Reinforcement of Rubber: Perspective on the Original Polymer Nanocomposite. Polymers.

[59] Huang Y., Gohs U., Müller M.T., Zschech C., Wießner S. (2019). Evaluation of Electron Induced Crosslinking of Masticated Natural Rubber at Different Temperatures. Polymers.

[60] Cox H.L. (1952). The elasticity and strength of paper and other fibrous materials. J. Appl. Phys..

[61] Hull D., Clyne T.W. (1996). An Introduction to Composite Materials.

